# A Logical Model of Conceptual Integrity in Data Integration

**DOI:** 10.6028/jres.108.034

**Published:** 2003-10-01

**Authors:** David Flater

**Affiliations:** National Institute of Standards and Technology, Gaithersburg, MD 20899-8264

**Keywords:** abstraction, data, integration, logic, semantics

## Abstract

Conceptual integrity is required for the result of data integration to be cohesive and sensible. Compromised conceptual integrity results in “semantic faults,” which are commonly blamed for latent integration bugs. A logical model of conceptual integrity in data integration and a simple example application are presented. Unlike constructive models that attempt to prevent semantic faults, this model allows both correct and incorrect integrations to be described. Imperfect legacy systems can therefore be modeled, allowing a more formal analysis of their flaws and the possible remedies.

## 1. Introduction

In the context of software, what is traditionally called “integration” is the engineering process that creates or improves information flows between information systems designed for different purposes. What actually flows between the systems is data, but what is critical to the business process is that all of the right data flows in the right form for the receiving system, and that the receiving system and the people who use it interpret the data correctly.

The term “conceptual integrity” was popularized in Ref. [1] to refer to a kind of consistency in system architecture that allows the system to become a cohesive, sensible whole. A similar kind of conceptual integrity is required for the result of data integration to be cohesive and sensible. Compromised conceptual integrity results in “semantic faults,” which are commonly blamed for latent integration bugs.

Most technical approaches to data integration fall squarely into one of two categories. There is the “global schema” category, where every schema is mapped into a common reference schema, and there is the direct translation category, where schemata are mapped directly to one another in a point-to-point fashion. Each category has widely recognized advantages and disadvantages. Among these is the efficiency argument in favor of standardization (i.e., having a standard global schema): to link *n* different systems directly requires *n*^2^ – *n* one-directional mappings, but to link them via a global schema requires only 2*n*.

It is sometimes claimed that direct translation allows for better conceptual integrity on a technical level (ignoring the human factors of dealing with *n*^2^ – *n* different translations) because one can translate only what is necessary for communication and ignore anything that is conflicting but irrelevant. However, after discussion of this point in the Automated Methods for Integrating Systems [2] project, it was realized that such a translation *implies* a certain “integration schema” which, regardless of whether it is written down or only in the mind of the integrator, is nevertheless equivalent in its impact on conceptual integrity to having used a global schema.

With that perspective, it should be possible to create an abstract model of conceptual integrity that is independent of the technical approach that is chosen for data integration. This paper documents such a model. The goal is not to provide another method for maintaining conceptual integrity, but to provide a logical model of conceptual integrity itself, capable of describing both correct and incorrect integrations resulting from whichever methodology is employed.

## 2. Related Work

Reference [3] contains a model that is similar in approach yet foundationally different from the one in this paper. Modal logic and logical properties are used to build a detailed model of identity, and the ramifications for correct and incorrect subsumption relationships are examined. Possible analogies to Ref. [3] are discussed in Sec. 7.

An alternate view on the issues discussed in this paper can be found in work having to do with context logic, which traces back to McCarthy [4,5]. A concise discussion of the application of context logic to information integration is given in Ref. [6]; see also the “Integrating Databases” example in Ref. [7], multicontext (MC) systems as described in Ref. [8], and the context-based schema analysis in Ref. [9]. The relationship between the view of this paper and the context logic view is explored in more detail in Sec. 8.

Logic-based approaches to schema integration, e.g., Refs. [10] and [11], are constructive methods intended to maintain conceptual integrity; i.e., they assume that one works within the method when integrating schemata and that the intensional definitions constructed by the modeler are complete and logically sufficient. A loss of conceptual integrity within the model would be indicated by the presence of a logical contradiction, which would render any subsequent logical inferences meaningless. Consequently, these logic-based approaches to schema integration are not ideal for describing and analyzing potentially imperfect integrations resulting from other methodologies.

The views of class and abstraction in this paper partially reflect ideas appearing in Ref. [12].

## 3. Logical Notation

Belief and time are critical to integration. Integration is performed at a point in time and from a point of view. Appropriately, this paper uses symbols from temporal modal logic as well as a “doxastic” modal (pertaining to belief).

The following descriptions are quoted from Ref. [13].

□ It is necessary that …
◊ It is possible that …
G It will always be the case that …
F It will be the case that …
H It has always been the case that …
P It was the case that …
Bx x believes that …

∧, ∨, and ~ are the conjunction, disjunction, and negation operators of classical logic. ≡ represents logical equivalence; i.e., the left hand side and the right hand side necessarily have the same truth values.

Let *p*, *q*, and *r* represent arbitrary logical sentences. The modals □ and ◊ relate to each other as follows.

□p≡~⋄~p (1)

⋄p≡~□~p (2)

□~p≡~⋄p (3)

⋄~p≡~□p (4)

The temporal modals have similar relations.

Fp≡~G~p (5)

Pp≡~H~p (6)

A distinction is made between *material implication*, represented by the symbol ⊃, and *strict implication*, represented by →.

*Material implication* is the truth-functional connective of classical logic.

p⊃q≡~p∨q (7)

*Strict implication* expresses the stronger statement that the consequent *necessarily* follows from the antecedent (i.e., is logically entailed or true by definition) [14].

p→q≡□(p⊃q) (8)

Strict implication must not be confused with *relevant implication* as used in relevance logics [15] or otherwise conflated with “relevance.” Relevance is not required. It is acceptable (albeit unhelpful) that a tautology (a necessarily true sentence) is strictly implied by any sentence whatsoever.

□q→(p→q) (9)

The need to distinguish between material implication and strict implication arises here because of time. As new individuals are created, strict implications that are true remain true, but the truth values of material implications can change. For example: over time, more people will be born; by definition, they will all be mortal (i.e., being a person strictly implies mortality); however, the fact that all people live on Earth might not remain true (even though “being a person materially implies living on Earth” holds at the present time). If the universe of discourse were static, the distinction would be moot: if an implication happened to be true for the universe as it was, then it would suffice for all discussions about that universe.

The reader is encouraged to consult Refs. [13], [14], and [16] regarding the spectrum of modal and temporal logics that are distinguished by the axioms accepted. Reference [16] identifies a series of systems that build on the following axioms (paraphrased):

(SL = Sentential Logic) Every theorem of classic sentential logic is a theorem.
(MP = *Modus Ponens*) If *p* is a theorem, and *p* ⊃ *q* is a theorem, then *q* is a theorem.
(Nec = “rule of necessitation”) If *p* is a theorem, then □ *p* is a theorem.
(◊) ◊*p* is defined as ∼□∼*p*.

The above axioms are accepted here. System T is formed by adding the following two axioms, which are also accepted here.

□(p⊃q)⊃(□p⊃□q) (10)

□p⊃q (11)

It follows that

p⊃⋄p (12)

In addition, the following axioms are accepted. (*N.B.*, since these are theorems, then by the rule of necessitation and the definition of strict implication, their counterparts using strict implication are also theorems.)

Gp⊃Fp (13)

Hp⊃Pp (14)

p⊃GPp (15)

p⊃HFp (16)

□p⊃G□p∧H□p (17)

G(p⊃q)⊃(Gp⊃Gq) (18)

H(p⊃q)⊃(Hp⊃Hq) (19)

## 4. Model

### 4.1 Foundation

A schema is a set of identified collections or groupings. Those collections would be called classes in an object-oriented system, tables in a relational system, concepts in a knowledge-based system, etc. For readability, the word “class” will be used for a collection or grouping, and the word “individual” will be used for that which is grouped (instance, tuple, etc.).

Let α, β, γ, and δ range over classes, let *a* range over individuals, and let *A* range over properties. A Boolean model of properties is assumed. *Aa* is true if and only if individual *a* has the property *A*. Define *Ā* to be the negation of *A*.

$$\bar{A}a\equiv \sim Aa$$ (20)

$$Aa\vee \bar{A}a$$ (21)

$$\sim (Aa\wedge \bar{A}a)$$ (22)

Define N(α) as the set of properties that are *necessary* for membership in α.

A∈N(α)≡a∈α→Aa (23)

Define O(α) as the set of properties that are *possible* for (consistent with) membership in α.

A∈O(α)≡⋄(a∈α∧Aa) (24)

It is assumed that one will abstain from defining classes that are *necessarily* empty (also known as “incoherent” classes).

These more intuitive theorems about N and O then follow:

a∈α∧A∈N(α)→Aa (25)

$$A\in N(\text{α})\to \bar{A}\notin N(\text{α})$$ (26)

a∈α∧Aa→A∈O(α) (27)

$$A\in O(\text{α})\to \bar{A}\notin N(\text{α})$$ (28)

$$A\notin O(\text{α})\to \bar{A}\in N(\text{α})$$ (29)

$$A\in N(\text{α})\to A\in O(\text{α})\wedge \bar{A}\notin O(\text{α})$$ (30)

It is possible for both a property and its negation to appear in O, with neither appearing in N.

As with □ and ◊, it would suffice to have only N or only O, but having both allows for more intuitive formulations.

Importantly, it is assumed that membership in classes is primitive. N contains properties that are *necessary* for membership in a class [as stated in Eq. (25), membership in a class *does* strictly imply that the individual has the necessary properties], but they are not logically *sufficient* to determine the membership. Classes are not necessarily characterized by a set of properties: in general, *A* ∈ N(α) ⊃ *Aa* does not strictly imply *a* ∈ α. Ideally, the “intent” of a class would be reflected by the properties in N, but the *extent* (its membership) is what is assumed to be known.

### 4.2 Subsumption

N and O display a symmetry with respect to subsumption.

(a∈γ→a∈δ)∧A∈N(δ)→A∈N(γ) (31)

(a∈γ→a∈δ)∧A∈O(γ)→A∈O(δ) (32)

It follows from Eqs. (30) and (32) that a property that is necessary in a subclass must be consistent with the superclass:

(a∈γ→a∈δ)∧A∈N(γ)→A∈O(δ) (33)

It is not always obvious that defining a subclass has ramifications for the meaning of the superclass, but it is true nonetheless. If someone defines a subclass Six-Legged-Dog, and the subclass is not *necessarily* empty, it follows that having six legs is consistent with being a dog. This may greatly surprise the person who defined Dog originally, but such is the kind of detail that one needs to know in order to perform a correct integration.

### 4.3 Conceptual Integrity

Let *S* and *T* represent different schemata (e.g., the data models implemented in two separate software systems). *S* and *T* do not share individuals or classes; however, they are discussed in terms of the same properties, all of which are within the same logical context.

For α of *S* and β of *T*, the simplest form of integration is a partial “instance map” from members of α to members of β. Let M(*a*) for *a* ∈ α represent the *analog* of *a* (its image under M, if such exists) in β. To maintain conceptual integrity, the following condition must hold for all *A*:

a∈α∧M(a)∈β∧Aa⊃A∈O(β) (34)

To paraphrase: if an individual with a given property is mapped to an analog in β, then that property must be consistent with membership in β. It is not necessary that the analog possess that property if the negation of that property is also in O(β); nor is it necessary that every individual in α have an analog.

## 5. On Abstraction

Data models as we know them are abstractions, and so are the mental models of the people who construct them. By definition, an abstraction of a thing or event is not identical to the thing or event itself and does not have all of its properties. Moreover, any documented model is at best an approximate expression of a mental model [17], and different data modelers think about different properties even when they believe that they are modeling the same thing. These differences can lead to a wide variety of conflicts [9,18,19].

Every thing or event has an unbounded set of properties. A data modeler tries to settle on a finite set of properties that suffices for a particular application. But when two applications are integrated, the properties that were captured in documented models may no longer suffice.

Consider the acquisition by a leading manufacturer of 100 % recycled content corrugated boxes of a relatively obscure company that makes biodegradable bubble wrap. The box company has a technically superior customer database, but the bubble wrap company has some specialized applications integrated with its own database that would be expensive to change. So it is decided to use the box company’s database as the primary one and just replicate the data in the other database for the sake of the specialized applications. This seems to work, and environmentally conscientious mail-order operations the world over rejoice that they can now obtain boxes *and* bubble wrap through the far-reaching distribution network of the former box company. Then disaster strikes. The box company’s best customer, the John Q. Fictional Company of Hanover, calls to complain that the bubble wrap they ordered never arrived. Investigation reveals that the order in question was shipped to the John Q. Fictional Company of Anchorage, a new customer who had simply ordered a small number of corrugated boxes. It turns out that one of the bubble wrap applications was written to key by company name, so it retrieved the wrong John Q. Fictional Company from the merged database and propagated the error.

It is important to understand that the box company’s database was not “wrong” to allow two companies to use the same name. Prior to the integration, it made no difference. The box company’s applications did not rely on names being unique. Neither was it “wrong” for the bubble wrap application to key by company name. Prior to the integration, company names were unique within the bubble wrap customer base. The problem was *created* by the integration.

Abstractions themselves have abstractions, and these are not immune to integration faults. For example, a common abstraction of time-of-day (itself an abstraction) constrains seconds to range between 0 and 59. An artifact that embodied this assumption might integrate successfully with many applications and operate for years without failure. However, cognizant data modelers are aware that an extra second—a “leap second”—is occasionally inserted into the Coordinated Universal Time (UTC) time scale to keep it within ±0.9 s of the Universal Time (UT1) astronomical time scale [20]. The time-of-day corresponding to the leap second is represented as 23:59:60. So if the artifact that constrains seconds to the range 0 to 59 is integrated with any that propagate leap seconds, it might fail all of a sudden one New Year’s.

For any given abstraction, it is possible to construct an integration scenario in which a failure will occur because of some property that was not explicitly modeled. The need for the abstractions of *S* (e.g., customer name according to the box company) to take an explicit stance with respect to properties that are relevant in *T* (e.g., uniquely identifying a customer) only *arises* when integration is attempted. Yet by virtue of numerous undocumented and/or un-thought-about implementation details, any realizations of these abstractions in engineered artifacts such as software implicitly take stances with respect to all properties. Simplistically, one could say that when confronted with new properties, either they work or they don’t.

## 6. Semantic Faults

“Semantic fault” is an informal term that can now be understood formally to mean a violation of the condition expressed in Eq. (34).

This section demonstrates how the semantic fault stories of Sec. 5 can be formalized. However, it is not necessarily the case that all semantic faults would emerge in exactly the same way.

Logical statements below describe the behaviors of the engineered artifacts as built unless preceded by the doxastic qualifier Bi (signifying a belief of the integrator, i).

Consider the following:

A∈O(β) (35)

a∈α⊃Aa (36)

$$\text{Bi}(a\in \text{α}\to Aa)\vee \text{BiG}(a\in \text{α}⊃Aa)\vee \text{Bi}(\bar{A}\in O(\text{β}))$$ (37)

The integrator builds a complete mapping from α to β,

a∈α⊃M(a)∈β (38)

and the integrated system functions normally. Now assume that at some future time, individual x will be born such that

$$F(x\in \text{α}\wedge \bar{A}x)$$ (39)

Assuming that *a* ∈ α ⊃ M(*a*) ∈ β remains true, conceptual integrity, Eq. (34), will then demand

$$F(\bar{A}\in O(\text{β}))$$ (40)

which is not guaranteed. If the behavior of the engineered artifact is instead described by *A* ∈ N(β), then $G\left(\bar{A}\notin O\left(\beta \right)\right)$; the condition of Eq. (34) will be violated, and there will be a semantic fault.

With the bindings shown in Table 1, the above models the examples described in Sec. 5. In the first example, individual x is the customer name “John Q. Fictional, Inc.;” conceptual integrity fails because that name is associated with more than one customer, which is inconsistent with the customer name class as projected from the bubble wrap application. In the second example, individual x is the time-of-day value 23:59:60; conceptual integrity fails because that time-of-day has seconds outside the range 0…59, which is inconsistent with the time-of-day class as represented in the failing application.

## 7. Analogies to Ref. [3]

Reference [3] defines *essential*, *rigid*, *non-rigid*, and *anti-rigid* as properties of properties. The definitions are made in terms of properties, individuals, and instances of properties (i.e., individuals that have that property). Classes as such are subsumed by properties that completely characterize them.

- A property is *essential* to an individual if and only if it necessarily holds for that individual at every possible time in every possible world.
- A property is *rigid* if and only if, necessarily, it is essential to all of its instances.
- A property is *non-rigid* if and only if it is not rigid.
- A property is *anti-rigid* if and only if it is not essential to any of its instances.

The concern whether a property is essential to an individual is different from the concern whether a property is necessary for membership in a class. These two concerns may become inextricable when classes are defined intensionally (when the possession of a given set of properties strictly implies class membership), but they do not when class membership is primitive. This divergence makes it difficult to construct valid analogies between the content of this paper and that of Ref. [3], despite apparent similarities.

Returning to the definitions of Sec. 4.1, one could draw limited, perhaps strained, analogies. Given a class α and a property *A*, one could say that *A* is *rigid* within α if *A* ∈ N(α), *non-rigid* if $\bar{A}\in O(\text{α})$. But class-centered analogs to *essential* and *anti-rigid* would require an intensional viewpoint.

## 8. Relationship to Context Logic

In works about context logic it is common to use the notation ist(*c*, *p*) to signify that proposition *p* is true in the context *c* [5]. That convention is adopted here.

Context is broadly interpreted and can be used in lieu of many specialized modals. One can identify contexts corresponding to spans of time, a particular person’s beliefs, etc.

In the case of data integration, it is natural to identify contexts corresponding to the schemata being integrated and then make assertions about what is true of various classes in those contexts. For example, if C_1_ is the context of a leap seconds cognizant time service, C_2_ is the context of some application, and τ is “the” time-of-day class, then one would write the following, or something equivalent:

$$\text{ist}(C_{1},\;\text{SecondsMayExceed}59(\text{τ}))$$ (41)

$$\text{ist}(C_{2},\;\sim \text{SecondsMayExceed}59(\text{τ}))$$ (42)

“The” time-of-day class is an abstraction inherited from a common context, such as a global schema. Its specializations in contexts C_1_ and C_2_ disagree with respect to the predicate SecondsMayExceed59. If the reference to a common context is eliminated, then there is no basis for discussion of whether the classes in C_1_ and C_2_ are compatible.

The model presented in this paper does not require that classes from a common context be made explicit. It does rely on the assumption that properties have equivalent meanings in the contexts of the systems being integrated. However, this is analogous to the assumption that predicates such as SecondsMayExceed59 have the same meaning in multiple contexts.

Of course, there is nothing to prevent one from making logical statements about predicates in different contexts; e.g.,

$$\text{ist}(C_{2},\;\text{ValidTimestamp}(a)⊃\text{Seconds}(a)<60)$$ (43)

But the problem repeats itself. Unless Seconds has a common interpretation, nothing has been gained by contextualizing ValidTimestamp.

Ultimately, to make comparisons between two contexts, it is necessary to have *some* common vocabulary with which to conduct the discussion. The problem can be moved around but cannot be eliminated. As always, “there is no silver bullet” [1], but a change in viewpoint can sometimes help. The goal is to move the problem to where it causes the least amount of damage.

## 9. Conclusion

A logical model of conceptual integrity in data integration and a simple example application have been presented. Unlike constructive models that attempt to prevent semantic faults, this model allows both correct and incorrect integrations to be described. Imperfect legacy systems can therefore be modeled, allowing a more formal analysis of their flaws and the possible remedies.

Future work to extend the model could focus on better treatment of several issues that were glossed over or minimized.

- The important temporal dimension of conceptual integrity could be explored in more detail and modeled more precisely.
- The abstractions implicit in the act of integration (pieces of an implicit “integration schema”) could be analyzed. A partial mapping from members of α to members of β suggests an abstraction from α and β that describes that part of the population that is “interesting” for the integration. A variant of formal concept analysis [21] may be applicable, as may currently evolving work on describing relations between ontologies [22].
- “Fuzzy” properties (i.e., where *Aa* is neither entirely true nor false, or is not known with certainty to be true—the different interpretations have different ramifications) could be explored. Additional analysis is needed to determine whether they add value. An infinite set of Boolean properties may render fuzzy properties redundant: if *Aa* is only “sort of” true, then it may be possible to derive a narrower “sub-property” that is fully true and another one that is fully false. On the other hand, it would be ill-advised to accept philosophical *vague* properties [23,24], which defy objective evaluation.

## Figures and Tables

**Table 1:** Bindings for Sec. 5 examples

	Boxes	Time
α	Customer name as projected from box company’s database	Time-of-day as delivered by leap seconds cognizant time service
β	Customer name as projected from bubble wrap application	Time-of-day as represented in some application
*A*	Associated with exactly one customer	Has seconds in range 0…59
*Ā*	Not associated with exactly one customer	Does not have seconds in range 0…59
